# Vaccine confidence: the keys to restoring trust

**DOI:** 10.1080/21645515.2020.1740559

**Published:** 2020-04-16

**Authors:** Selim Badur, Martin Ota, Serdar Öztürk, Richard Adegbola, Anil Dutta

**Affiliations:** a EM, Vaccines Scientific Affairs and Public Health, GSK, Istanbul, Turkey; b EM, Vaccines Scientific Affairs and Public Health, GSK, Wavre, Belgium; c EM Central, Vaccines Medical, GSK, Istanbul, Turkey; d Immunisation & Global Health Consulting, RAMBICON, Lagos, Nigeria; e Vaccines R&D Medical, GSK, Wavre, Belgium

**Keywords:** Health education, health knowledge, immunization, vaccination coverage, vaccination refusal, vaccine hesitancy, parental psychology, public trust, social media

## Abstract

During the 20^th^ century, the discovery of modern vaccines and ensuing mass vaccination dramatically decreased the incidence of many infectious diseases and in some cases eliminated them. Despite this, we are now witnessing a decrease in vaccine confidence that threatens to reverse the progress made. Considering the different extents of low vaccine confidence in different countries of the world, both developed and developing, we aim to contribute to the discussion of the reasons for this, and to propose some viable scientific solutions to build or help restore vaccine confidence worldwide.

## Introduction

Vaccination is one of the most important success stories of modern-day medicine, averting over 5 million deaths worldwide every year from 2010 to 2015. During the 20^th^ century, mass vaccination resulted in dramatic decreases in the incidence and morbidity of many infectious diseases. In most countries, a number of infectious diseases have been mitigated and, in some cases, have been eliminated due to routine vaccination.^1,2^ One human disease – smallpox – has been completely eradicated by the widespread use of specific vaccines, and poliomyelitis is on track to be the next.^1,3^ Every year vaccination prevents 2.7 million cases of measles, 2 million cases of neonatal tetanus, 1 million cases of pertussis, 600,000 cases of paralytic poliomyelitis and 300,000 cases of diphtheria.^3^

In addition to directly preventing disease in vaccinees, vaccination indirectly reduces the likelihood of disease transmission. Unvaccinated individuals have a reduced risk of contracting the infection once a critically high proportion of the population has been immunized, a phenomenon known as *herd immunity*.^4,5^ Vaccination can also reduce antibiotic use,^6,7^ thereby having a direct effect on antimicrobial resistance, as shown by the pneumococcal conjugate vaccines that have substantially reduced the incidence of disease caused by antibiotic-resistant strains. In addition, experience with rotavirus and pneumococcal diseases has further shown that effective use of vaccines can reduce diagnostic and treatment costs, numbers of ambulatory care visits, medical interventions, and hospitalizations.^8–13^ It can also prevent nosocomial infections, and, in other cases, indirectly prevent some types of cancer.^14^ Dual vaccination with pneumococcal and influenza vaccines protects older adults with chronic illness from hospitalization for certain respiratory, cardiovascular, and cerebrovascular diseases, thereby reducing the risk of intensive care unit admission and death.^15,16^

In addition to being effective in reducing disease prevalence and associated mortality, vaccination provides the benefit of averting costs of medical care. Ozawa et al.^17^ examined the return on investment associated with achieving projected coverage levels for 10 antigens in 94 countries. They estimated that universal vaccination would yield a net return about 16 times greater than costs over the decade.^17^ A further positive outcome of vaccination is the reduced workload for healthcare providers (HCPs) resulting from averted illness. This is particularly important in developing countries with a much lower density of HCPs, who are often overwhelmed by the number of patients requiring their attention. The assessment of the value of vaccines and vaccination needs to go beyond the direct impact on health and healthcare by considering the wider impact on societal and household economic well-being.^18^

Because of all these proven benefits and the well understood importance of vaccination, suitable vaccination programs have been implemented around the world. Despite this, many countries still have vaccination coverage rates below that stipulated by the World Health Organization (WHO). These programs have therefore been focused on assuring that high vaccination coverage is achieved in all regions of the world to grant everyone protection. Unfortunately, these efforts are being undermined by a growing anti-vaccination movement. The hesitancy of individuals to have themselves and their children vaccinated can have a profound impact not only on their own health, but also on public health in general. Indeed, some vaccine-preventable diseases (VPDs) have reemerged in both developed and developing countries.^19–21^ The resurgence of infectious diseases that are currently under control or have already been eradicated is a real danger.^22^ Therefore vaccine hesitancy (VH) has been included in the WHO list of global health threats for 2019.^23^

**Figure 1.**
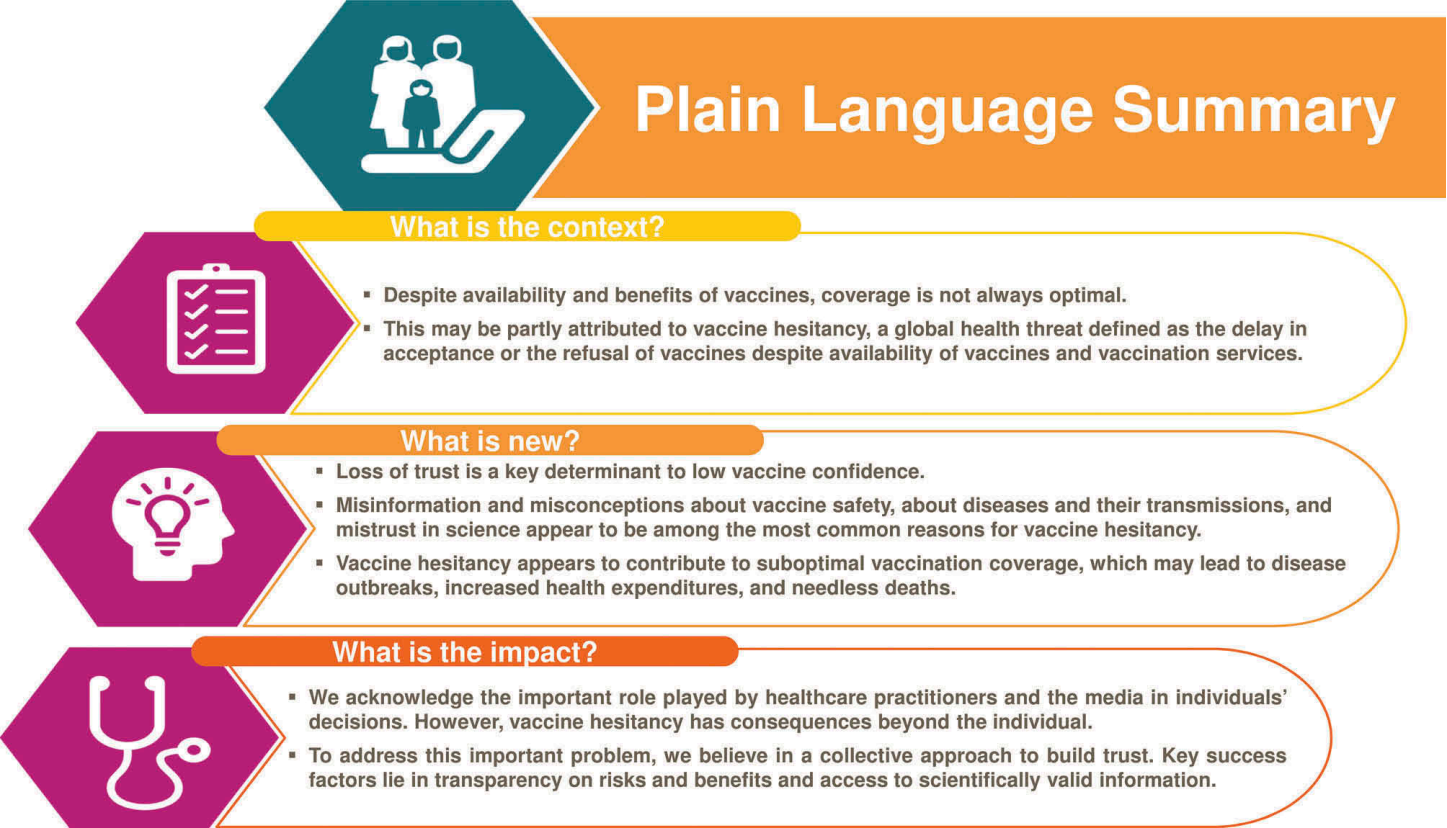
Plain language summary.

VH is defined by the WHO Vaccine Hesitancy Working Group as the ‘delay in acceptance or refusal of vaccines despite availability of vaccine services’, and is a complex behavioral phenomenon ‘influenced by factors such as complacency, convenience and confidence’. VH is context-specific, varying across time, vaccines, places, and populations within a place.^24^ Hence, a considerable number of children have delayed vaccination secondary to a wide range of causes.^25^ With a focus on childhood vaccination, the present article aims to contribute to the discussion on causes and implications of the low vaccine confidence in developed and developing countries and to propose viable evidence-based solutions to improve vaccine confidence (Figure 1).

## Current situation and grounds for low vaccine confidence

### Misinformation and hoaxes

Misinformation and lack of access to balanced and accurate information is a major contributor to low vaccine confidence. The impact of misinformation has grown with the easy and rapid proliferation of unfounded and invalid information through mass communication media.^26–29^ In developed countries, the internet and social media have played a central role in the advancement of anti-vaccination movements and in shaping vaccination decision-making.^26,28,30^ An analysis of vaccine criticism on the internet,^31^ found that websites critical of vaccines argue that vaccines cause illness, claim conventional medicine is wrong, and make emotional appeals and allegations about conspiracies, civil liberty violations, totalitarianism, and immorality, whilst encouraging alternative medicine.^31^ In a recent commentary, Heidi J Larson, the director of the Vaccine Confidence Project at the London School of Hygiene and Tropical Medicine, stated that there was a ‘deluge’ of misinformation on social media that ‘should be recognized as a global public-health threat’.^32^

Misinformation about vaccines abounds across all settings. The mode of propagation may differ, but fabricated information (hoaxes) about vaccines may gain traction when it is spread by opinion leaders. For example, in Cameroon, in 1990, one such rumor was spread that a tetanus vaccine was used to sterilize girls and women.^33^ Five years later, vaccination rates against tetanus declined to as low as only 13%.^33^ In Nigeria, in 2003, rumors that an oral polio vaccine (OPV) was an American conspiracy to sterilize Muslim girls and spread human immunodeficiency virus resulted in the suspension of OPV use in five northern Nigerian states.^34^ Consequently, wild poliovirus cases in the country increased fivefold between 2002 and 2006, causing a nationwide epidemic.^34^ Moreover, the Nigerian strains of poliovirus were transmitted across Africa and beyond and re-infected previously polio-free countries.^34,35^ In both cases, the rumors were endorsed by local opinion leaders.^33,35^ Over 40% of the worldwide wild polio virus cases recorded in 2011 and 2012 occurred in the common epidemiological block of Pakistan and Afghanistan.^36^ Pakistani parents who had refused OPV in a region with nine annual OPV campaigns attributed their refusal mainly to the perception that the vaccine was associated with birth control and disapproval of religious leaders.^36^ Pakistan and Afghanistan are the only two countries where polio has still not been eradicated.^37–42^

### Loss of trust and risk communication challenges

**Figure 2.**
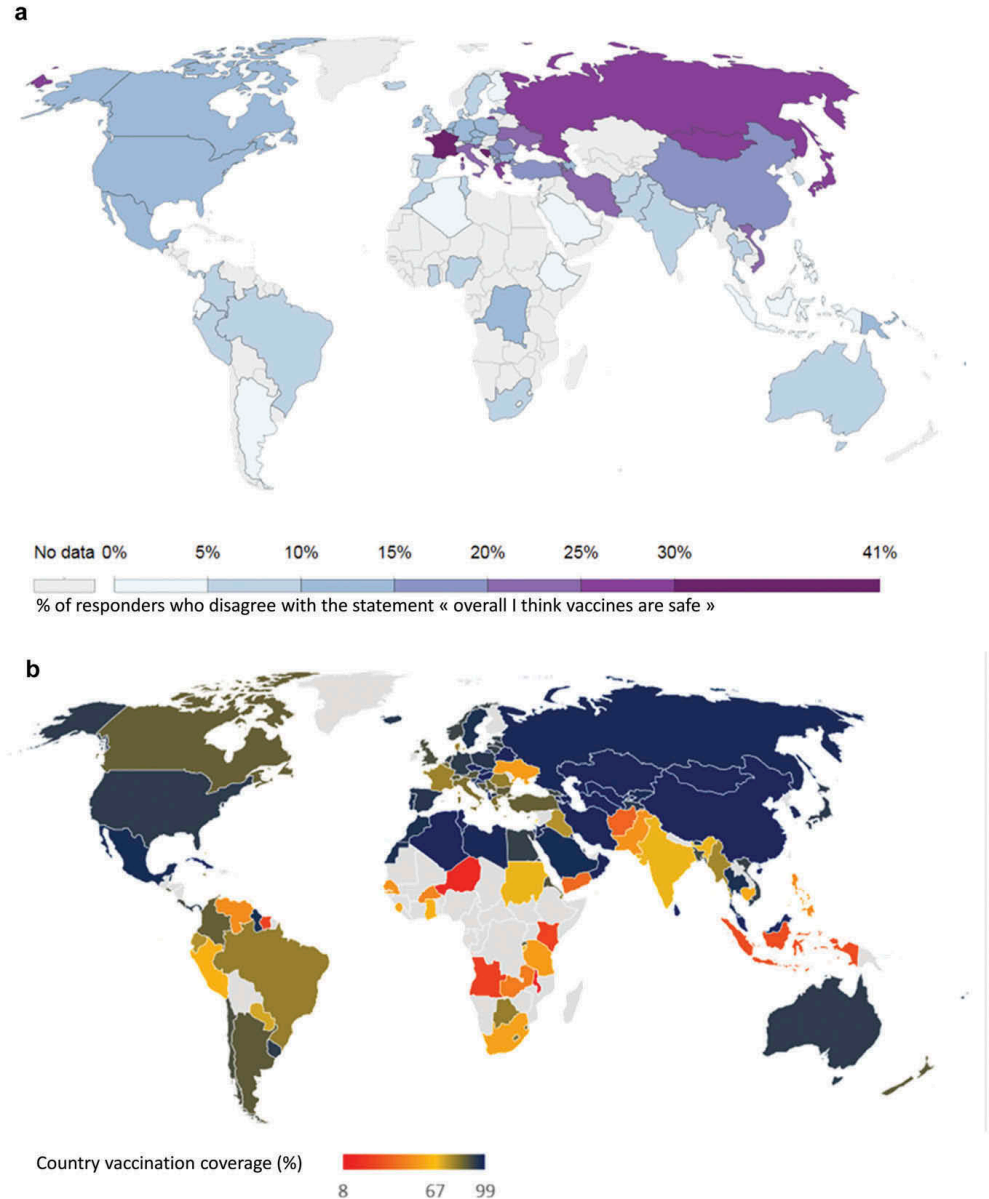
Perception of vaccine safety and measles vaccination coverage worldwide in 2015. Confidence in vaccination may be a determinant to vaccination rates, although it is not the only factor. A, Levels of confidence in vaccine safety (2015), Adapted from *Our world in Data*,^43^ based on a study from Larson et al., 2016.^44^ B, Measles (MCV2) vaccination coverage (2015). Source: WUENIC.^45^

Several unfortunate events have negatively influenced public opinion and trust in vaccination and vaccine safety (Figure 2), both in developped and developing countries, as exemplified below. When vaccine safety issues occur, they can be amplified and misrepresented leading to scaremongering behaviors and misinformation on social media. Furthermore, sub-optimal handling of difficult situations may decrease vaccine confidence and thereby adversely affect vaccination programs.

In developing countries, such as Pakistan, polio eradication has been consistently undermined not only by religious extremism spreading rumors claiming that the vaccination is part of a Western conspiracy to sterilize Muslim persons, but also by the public’s loss of trust in the vaccination campaigns.^36,38,39^ This loss of trust was fueled by a 2011 CIA plot that used a vaccination campaign to track down Osama bin Laden. As a result, health workers and vaccinator team members have been killed in numerous attacks from 2016 to 2019.^40–42^

Also in developed countries, such as Japan, uncertainty and mistrust was triggered by the health authorities ambiguous choice to suspend the recommendation for routine human papillomavirus (HPV) vaccination following allegations of adverse effects, while allowing the voluntary vaccination.^46,47^ A year later, the authorities concluded that the adverse reactions were not causally related to the HPV vaccine.^46,48^ However, they did not revoke the suspension of routine recommendation, thus exacerbating public confusion and uncertainty. While this was happening, unverified information spread through online media which reached and influenced a wider, worldwide audience.^46^ Deaths from cervical cancer have increased by 9.6% in Japan in the past 10 years, and the HPV vaccination rate among girls eligible for vaccination dropped from 70% in 2013 to <1% in 2014.^48,49^ Still today, the Japanese health authorities have not resumed their recommendation for HPV vaccination. In Europe, public trust in the authorities was challenged and low adherence to influenza vaccination campaigns was recorded following the 2009 H1N1 pandemic.^50,51^ The influenza outbreak had far fewer consequences than initially anticipated while the national budgets spent were considered by the Council of Europe to be disproportionate and unjustified.^51,52^ Consequently, the definition for pandemic used by the WHO was subject to public controversy, and received wide-spread media coverage. Public trust in the authorities was challenged and low adherence to subsequent influenza vaccination campaigns was recorded.^50,51^

### HCP involvement

HCPs are the most frequent source of information regarding vaccination, and the interaction between patients or their parents and HCPs is at the core of maintaining confidence in vaccination.^53–55^ Not only the knowledge, but also the attitude of the HCPs, are important to convey trust. The investigators of a 2014 national United States (US) survey also suggested that high quality recommendations from HCPs encouraged vaccination-hesitant parents to accept vaccination.^56^ However, only one-third of the parents participating in the survey had received such high quality recommendation on HPV vaccination, and half of the parents had not received any recommendation for HPV vaccination.^56^ A 2015 systematic review of studies conducted in the US found that HCPs did not strongly endorse the need for vaccination in their communication with parents.^57^ Vaccinated HCPs are more likely to recommend vaccination to their patients than unvaccinated colleagues (57.5% vs. 43.8%, *P* <.001).^58,59^ Yet, recent surveys indicate low influenza vaccination rates across settings among HCPs, with 27% in two university hospitals in Ankara, Turkey, and 47.6% for US HCPs working in settings where vaccination was not required, promoted, or offered on-site.^59,60^ Among HCPs, reasons for not recommending pneumococcal vaccination included lack of knowledge about vaccinations benefits, fears of adverse events, and doubts about efficacy and safety.^59^ It was also shown that HCPs who are knowledgeable about vaccines were more confident and more likely to recommend vaccination.^58^ Educational interventions aimed at improving HCP knowledge and communication about vaccination improved HCPs’ knowledge and the vaccine uptake of HCPs and their patients.^58,61^

Studies have shown, however, that HCPs’ knowledge about vaccination issues is variable and that training in vaccinology is poor or non-existent in medical curricula in many countries.^62^ In a French hospital study, only 25% of HCPs were able to list correctly the three mandatory vaccines.^63^ Similarly, only 12.9% of Greek HCPs correctly named the vaccines recommended for them by the Ministry of Health.^64^ In an Australian hospital-based study, only 9.8% of HCPs were able to correctly identify the vaccines recommended for HCPs.^65^

### Complex dynamics of immunization

The ‘pathogen-host-vaccine’ triad can be complex. A small percentage of non-vaccinated persons are not infected during an outbreak while another small percentage of persons contract a disease despite being vaccinated against it. Therefore, some parents may question vaccine efficacy, but expecting 100% efficacy is not realistic. In rare instances, a vaccine may fail to mount the appropriate immune response, which may be due to handling errors,^66^ or genetic determinants in the host or in the pathogen. Today, the mechanisms by which host and pathogen gene polymorphisms may influence immune responses to vaccines are better understood, but this complexity can be difficult to communicate (Supplement Text Box 1).^67,103-112^ Furthermore, the mitigation of severity and duration of the disease is an important benefit of routine vaccination. Therefore, although vaccines do not always produce a full immune response, they may nevertheless lessen disease symptoms, as seen with influenza, pertussis and rotavirus vaccines.^68–70^ This protective effect should be emphasized, as it may not always have been fully appreciated by HCPs and the general population.^68^

### Healthism

An increasing number of parents in the developed world believe that a ‘natural lifestyle’ and better hygiene and sanitation will make diseases disappear and that acquiring immunity through having the disease is better. They describe children’s bodies as naturally perfect, and believe that the ways by which vaccines enter the body are unnatural. They thus feel capable of managing their children’s health without vaccines because of their closer-to-nature lifestyles.^71,72^ A significant proportion of such parents may be unaware of the severity of vaccine preventable diseases and their potential consequences.^71^ Another key aspect relates to the use of complementary or alternative medicine instead of vaccines, and some practitioners (homeopaths, chiropractors, and naturopaths) have a negative view of vaccination and advise their clients accordingly.^73,74^

### Skepticism toward science

The healthism attitude, but also the tendency to believe in conspiracy theories, might be explained by new patterns of behavior regarding scientific evidence: belief in science is decreasing and the willingness to accept nonscientific approaches is increasing all around the world.^75^ The postmodern era we are living in is going through ‘an assault on science’ – as quoted by Kuntz.^76^ In postmodernist thinking, scientists are not to be trusted and therefore people should control scientific research. Denialism has also been proposed as a term that might describe these attitudes.^77^ Denialists might put forward fake experts to support their theories, thus trying to discredit the work of established experts and researches.^77^ They also make selective use of flaws in isolated papers, and in doing so spread disbelief in all scientific data.^77^ A study examining attitudes toward society members among vaccine-skeptics and non-skeptics found that vaccine skeptics are less likely to consider others as equals.^78^ According to the authors, their finding should be taken into consideration when designing communication strategies for vaccines.^78^

## Consequences of low vaccine confidence

### Medical consequences of low vaccine confidence

In a community, the incidence of VPDs is directly related to the number of unvaccinated persons.^79^ The reemergence of infectious diseases such as measles and pertussis has been linked, among other factors, to an increase in the number of parents refusing to have their children vaccinated.^79^ There is also a significant correlation between infectious diseases outbreaks and geographic aggregation of vaccination refusals.^79^ Ultimately, vaccination refusal not only increases the likelihood of contracting infectious diseases for the unvaccinated individual but for the whole community as well.^79^ For this reason, there is a need to reach a certain proportion of vaccinated individuals in the target population to prevent the occurrence of outbreaks.^1^ Even a modest gap in vaccination protection will offer an opportunity for the infectious disease agent to proliferate and cause outbreaks.^80,81^

The decline in infectious disease incidence due to vaccination has created the impression that the diseases are becoming scarce and less harmful.^82^ As evidence of this, the American Academy of Pediatrics reported that the percentage of parents who refused some vaccines almost doubled between 2006 and 2013, and that about 1 in 5 parents requested to delay vaccination.^83^ In the case of pertussis for example, in many countries, fear of the consequences of the disease faded after achieving high vaccination rates over the years, and worries over vaccine safety gained attention.^82^ This loss of vaccine confidence can be described as a ‘tragedy of the commons’, as it resulted in lowered vaccination rates, with consequent disease resurgence.^84^ With respect to measles,^79,85^ in the Netherlands, a measles outbreak started in 2013, with most cases (91.7%) occurring in orthodox Protestants who opposed vaccination; almost all infected cases (96.5%) were unvaccinated.^86^ In 2018, there were over 80,000 cases of measles in 47 European countries; 61% of patients required hospitalization and 72 died.^87^ In early 2019, based on WHO data, measles case numbers were on the rise, with 40–700% increases reported in various regions.^88^ 2019 was also the year with the greatest number of measles cases in the history of the US since 1992: 1,282 cases (375 in 2018), mostly unvaccinated, in 31 states.^89^

### Economic consequences of low vaccine confidence

Vaccination is generally associated with cost savings. A study evaluating the measles national vaccination coverage among 29 European countries between 1998 and 2011 found that vaccination coverage and burden of disease had a significant negative correlation (−0.025, 95% confidence interval: −0.047 to −0.003).^90^ The cost of measles therefore decreases with increasing vaccination rates. There was a societal cost savings of USD 13.5 billion in direct medical costs and USD 68.8 billion in total societal costs for the US society with routine childhood vaccination.^91,92^

Low vaccine confidence imposes a high economic burden on society as a whole, causing high direct healthcare costs, indirect productivity losses, and public health spending in the healthcare sector. Even small reductions in vaccination coverage have substantial public health and economic consequences.^80^ It is estimated that a 5% reduction in measles, mumps, and rubella (MMR) vaccination in the US would add a cost burden of USD 2.1 million to the annual public sector’s healthcare expenditures.^80^

The unnecessary economic burden for society caused by low vaccine confidence was clearly shown by evaluating the economic burden of several past measles outbreaks.^93–97^ In 2011, the US experienced 16 measles outbreaks and the total economic burden on public health institutions was estimated to be in the range of USD 2.7–5.3 million.^98^ In 2008, a measles outbreak in San Diego, that originated from an intentionally unvaccinated seven-year-old boy, cost the community nearly USD 177,000, which included medical care provision to the confirmed cases, tracking of suspected cases, quarantining people, enhancing surveillance, and following up an infected infant and related contacts in Hawaii.^94,99^ In 2013, in New York, the largest measles outbreak since 1992 infected 58 mostly intentionally unvaccinated persons, and the direct cost for controlling the outbreak was nearly USD 400,000.^97^ In Ethiopia, from October 2011 to April 2012, a measles outbreak caused seven deaths among >5,000 infected children.^100^ The economic burden of this outbreak corresponded to over USD 750,000. The health sector cost for the treatment of each case was two times the national health expenditure per person in 2010.^100^

## Proposed solutions

There is no single intervention to restore public confidence in vaccination, especially across countries that have very diverse sociocultural, economic and geopolitical backgrounds. Nevertheless, different strategies have been formulated to encourage vaccination among individuals reluctant to receive vaccines and among parents concerned about the risk of vaccines causing their children harm. Medical associations, the pharmaceutical industry, health authorities on local, national, and international levels are responsible for improving or restoring vaccine confidence; and we discuss some of these strategies below.

### Regaining trust

#### Provision of valid information

**Figure 3.**
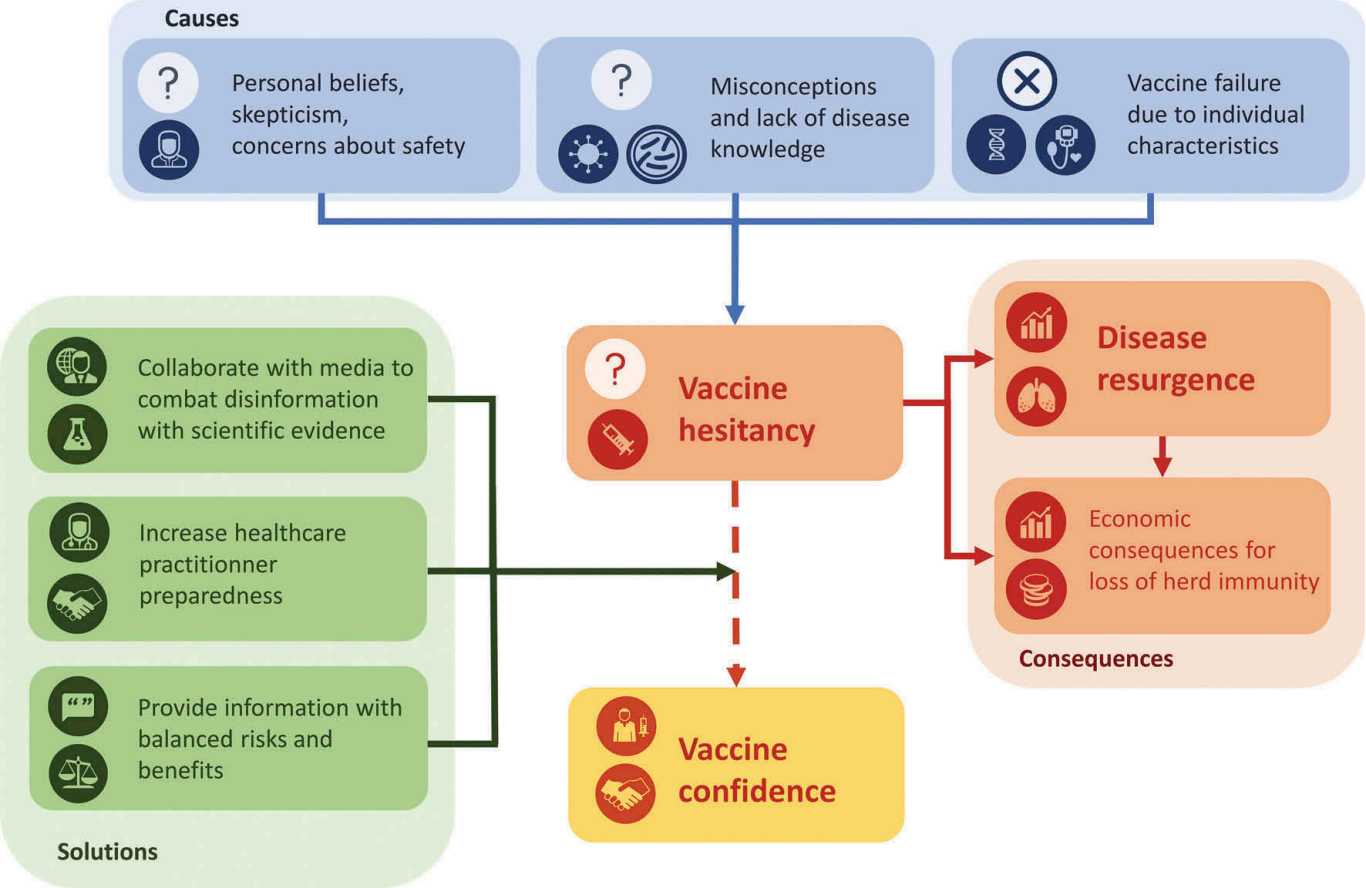
The vaccine hesitancy phenomenon: causes, consequences, and solutions.

One of the main reasons people make decisions based on unfounded information on vaccines is lack of trust in the healthcare system, public health organizations, the vaccine as a product, and the vaccine provider.^101,102,113^ Transparency about vaccine risks and benefits and increasing the pool of available material for public use is crucial in restoring and maintaining trust (Box 1, Figure 3). Access to credible information driven by scientific responsibility and integrity should be facilitated and ensured for the public, vaccine providers (HCPs, nurses, midwives), and health authority officials.

**BOX 1.** A model example of risk communication.

In 2015 in Denmark, HPV vaccination coverage decreased by 55% after reports of long-lasting pain and tiredness following vaccination, although there was no evidence for a causal relationship. A television documentary on a group of girls with adverse symptoms alleged to be related to their HPV vaccination was widely shared on social media. After this, other similar case-histories were published through various media sources.^103^ As media attention was increasing, public confidence declined, and vaccination uptake decreased.^103^ The Danish health authorities conducted a survey to better understand people’s concerns. They found that there was an important lack of information among parents, and that they needed to know the basic facts about HPV vaccines. A campaign was launched to communicate scientific facts. The health authorities used the media to promote their campaign, they opened a dialogue on Facebook with people who wanted to share their own experience and concerns, and they introduced their audience to the pyramid of evidence to help readers critically appraise studies on their own. The Danish authorities’ efforts were successful as reflected by increased vaccination uptake in the first few months following the launch of the HPV campaign.

The public should also be aware of the rigorous process in place for the scientific evaluation of vaccines, the strict regulatory requirements to obtain approval, life-cycle management, and the continuous assessment of the safety profile after approval.

The pharmaceutical industry could do more to address people’s concerns with information suited to different audiences, such as video summaries of scientific articles,^104^ and plain language summaries of clinical trials.

#### International and cross-sector partnerships

Several different international collaborations are monitoring the status of vaccine confidence across the globe, including Regional Technical Advisory Groups on Immunization (RITAG), Strategic Advisory Group of Experts (SAGE), Tailoring Immunization Programmes (TIP) which are initiatives of the WHO; The Vaccine Confidence project; and Gavi, the Vaccine Alliance. They provide information and/or recommendations on issues such as lack of vaccine confidence. Such collaborative efforts could expand to the provision of technical advice on operational plans for implementation by each country’s government.^22^ According to a report from the WHO-SAGE, interventions that are most successful in improving vaccine confidence are multi-component strategies, some of which could involve the collaboration of several different sectors. Multi-component interventions such as exposure to a community influencer (e.g., trained personnel or family/community members),^105^ or attendance to parent meetings in addition to receiving an information leaflet,^106^ could improve vaccine uptake in settings where initial vaccine confidence is low. Other effective interventions that involve cross-sector partnerships include the use of web pages with tailored messages, which may be effective in improving vaccine compliance among vaccine-hesitant parents,^107^ or the use of a mathematical modeling approach to shape a message describing potential consequences of reduced vaccination uptake, which have an impact on improving mothers’ opinion of childhood vaccines.^108^ Partnerships between health authorities on an international level with the education sector, social partners, and public and private healthcare sectors would optimize awareness-raising campaigns, produce modern tools to increase vaccine confidence, and provide support to poorer countries.

### Use of the media

The role of the media – including the internet – need not be negative. First, media could provide valuable resources and tools to physicians helping them to engage with their patients with personalized information, which is more effective than non-tailored information in improving compliance with recommended health behaviors.^109^ Furthermore, health authorities should be able to harness the power of social media and use it to monitor trends in public opinion and respond accordingly.^110,111^ Unfounded allegations should be countered with scientific evidence on the risks and benefits of vaccines.^112^ Raising positive voices, potentially using community influencers,^105^ and sharing a cross-sector unified message presenting vaccine acceptation as the social norm, while acknowledging and positively valuing those who do accept vaccination,^113^ could help combat misinformation and the growing distrust in science. Moreover, the health authorities should take lessons from successful past uses of media (Box 1),^114,115^ and proactively prepare a media communication plan ready to be implemented in the event of a safety issue.^112^ Such a plan should include preparedness of media-trained spokespersons, and development of different sets of information depending on the audience and the means of communication (i.e., tailor-made for radio, newspapers, television, or social media).^112^ However, communication planning must not be limited to crisis management,^112^ but should be ongoing, proactively providing messages directly targeting the most crucial public concerns, taking into account social and cultural characteristics as well as geographical location influences.^116^

### Increase HCP preparedness

An information exchange is required when recommending a vaccine to a patient or to a parent. During this process, the HCP has to explain the nature and purpose of vaccines, present the scientific evidence including the risks and benefits for the child and for the community. Transparency about VPDs, vaccines, testing, ingredients, potential side effects and funding reduces mistrust.

It is also important to communicate this information positively. Researchers have found that a presumptive approach (i.e., assuming parents will opt for vaccination), was more efficient in convincing parents to have their children vaccinated, rather than a participatory approach (i.e., asking open questions involving parental decision-making).^117,118^ Patients’ and parents’ questions should be answered, and their fears, preferences, and values should be respected, as well as their autonomy and freedom of choice.^119,120^ The information provided must be clear, reliable, and include the latest findings on VPDs, vaccine safety and efficacy from credible sources. To provide the evidence that is needed by the parents to make informed decisions without oversaturating them with information, it is important that HCPs develop their ability to listen to parents’ concerns and communicate clear and understandable messages. Such an approach is key in building trustful communication between parents and HCPs and is one of the principles of motivational interviewing.^120^ Using motivational interviewing techniques, the parents will receive information from the HCPs tailored to their needs, which actually helps them in resolving ambivalence about accepting vaccination.^120^

HCPs need support in fulfilling the requirements of this very important information process. HCPs with knowledge about the recommended vaccines and the respective VPDs are more likely to recommend vaccination.^58^ Training courses on current knowledge and new developments regarding VPDs, vaccines, and related recommendations should be offered for HCPs at all levels of experience, from medical students to practicing physicians. The training should include techniques to manage difficult conversations with hesitant individuals.^58^ Supplying HCPs with the communication tools to be used during this process is crucial. Moreover, as HCPs are often bound by time constrains, viable workload solutions should be implemented to allow the required time for the information exchange,^58^ such as reimbursement of HCPs who vaccinate children with overdue routine childhood vaccinations schedules.^121,122^ Lastly, involving HCPs in the process of developing vaccination recommendations would improve their understanding and engagement in implementing them.^58^

## Conclusions

It seems there is a loss of trust in health authorities and science, also in the public and private health sectors, and rapid global sharing of misinformation are leading to an increase in the number of people questioning vaccines and sometimes delaying or refusing vaccination. Individual acceptance of vaccination depends on knowledge about the risks and benefits of vaccines, but also on more complex determinants such as cultural, religious, emotional, and social factors.^123,124^

Incorporating the progress and results of vaccine research into successful vaccination programs, and replacing misinformation with evidence-based communication is crucial in improving vaccine confidence and requires a multidisciplinary, cohesive, targeted, and managed approach. HCPs are the front-runners in the process of vaccination and should be well prepared with the appropriate knowledge to address decreased vaccine confidence. Respecting individuals’ beliefs and lifestyles while providing all scientifically founded information, including risks and benefits of vaccination, is fundamental in helping people understand the rationale and benefits of vaccination for both the individual and communities. The success of any vaccination strategy is determined by the people’s confidence in the vaccination system and their trust in health authorities; otherwise, vaccination strategies might become counterproductive.^111,119^ Strengthening of the vaccination system to maintain optimum vaccination rates in the long run, through education, legislation, regulation, supply chain improvements, and more, should be the priority for all parties involved in the provision of healthcare and is crucial to Sustainable Development Goals formulated for 2015–2030.^125^

## Abbreviations

DTaP: diphtheria, tetanus toxoid, and acellular pertussis
DTP: diphtheria tetanus toxoids and pertussis
HCP: healthcare provider
HBV: hepatitis B virus
HLA: human leukocyte antigen
HPV: human papillomavirus
MMR: measles, mumps, and rubella
MS: multiple sclerosis
OPV: oral polio vaccine
RITAG: Regional Technical Advisory Groups on Immunization
PRR: pathogen recognition receptor
TLR: toll-like receptors
UK: the United Kingdom
US(A): the United States (of America)
VH: vaccine hesitancy
VPD: vaccine-preventable disease
WHO: World Health Organization
