# Supplementary material for: Vaccine confidence: the keys to restoring trust
Source: Hum Vaccin Immunother. 2020 Apr 16;16(5):1007–17. doi: 10.1080/21645515.2020.1740559 (PMC7227637; doi:10.1080/21645515.2020.1740559)
Supplement: Supplemental Material [file khvi-16-05-1740559-s001.docx]

# Supplementary material

## Text Box 1. Some genetic determinants responsible for vaccine failure

***Genetic determinants in the host****

*Hepatitis B*

Up to 10% of immunocompetent vaccinees do not respond with a protective antibody level. In the case of hepatitis B vaccination, risk factors such as obesity, heavy smoking or chronic renal failure have been described in addition to a genetic predisposition to a lower antibody response in certain human leukocyte antigen (HLA) class II haplotypes (HLA-DRB1, HLA-DQB1).^126^

*Mumps*

The HLA-DQB1 0303 allele was found to be associated with a lower specific antibody titer^127^ and the B62 supertype was suggestive of an association with mumps-specific higher lymph proliferation after the measles, mumps, and rubella (MMR) vaccine.^128^

*Measles*

HLA gene polymorphisms, genetic variations in cytokine, cell surface receptor, and toll-like receptor (TLR) genes are also associated with variations in antibody levels after measles vaccination.^129-131^ Genetic variation in major pathogen recognition receptors such as the measles virus cell entry receptors CD46 and SLAM, the virus attachment receptor DC-SIGN and also the anti-viral TLRs (TLR3, TLR 7 and TLR8) may significantly influence measles IgG antibody responses.^132^

*Hepatitis A*

A lower number of non-responders have been found for vaccines, of which 2% developed no specific antibodies after primary vaccination. The level of expression of HAVcr-1 on CD4 T cells correlated significantly with the antibody response.^133^

***Genetic determinants in the pathogen***

Hepatitis B virus (HBV) mutations involving the ’a’ determinant region of HBV surface antigen – also known as ‘escape mutants’ – might be associated with vaccine failure in some infants.^134^

*Although genetic pre-vaccination screening is possible, it may be unpractical and costly in the context of universal mass vaccination.^135^
